# Changes in the Expression and Functional Activities of C-X-C Motif Chemokine Ligand 13 (*CXCL13*) in Hyperplastic Prostate

**DOI:** 10.3390/ijms24010056

**Published:** 2022-12-21

**Authors:** Daoquan Liu, Mingzhou Li, Xun Fu, Shu Yang, Zhen Wang, Jianmin Liu, Yan Li, Yongying Zhou, Pengfei Ren, Yuhang Guo, Xinghuan Wang, Michael E. DiSanto, Ping Chen, Xinhua Zhang

**Affiliations:** 1 Department of Urology, Zhongnan Hospital of Wuhan University, Wuhan 430071, China; 2 Department of Urology, Affiliated Hospital of Zunyi Medical University, Zunyi 563000, China; 3 Department of Surgery and Biomedical Sciences, Cooper Medical School of Rowan University, Camden, NJ 08103, USA

**Keywords:** benign prostatic hyperplasia, *CXCL13*, fibrosis, inflammation, proliferation, epithelial–mesenchymal transition

## Abstract

Background: C-X-C motif chemokine ligand 13 (*CXCL13*), a member of the CXC subtype in chemokine superfamily, affects numerous biological processes of various types of cells and the progress of a great number of clinical diseases. The purpose of the current study was to reveal the internal mechanism between *CXCL13* and benign prostatic hyperplasia (BPH). Methods: Human serum, prostate tissues and human prostate cell lines (BPH-1, WPMY-1) were utilized. The effect of recombinant human CXCL13 (rHuCXCL13) protein and the influences of the knockdown/overexpression of *CXCL13* on two cell lines were studied. Rescue experiments by anti-CXCR5 were also conducted. In vivo, rHuCXCL13 was injected into the ventral prostate of rats. Additionally, a tissue microarray of hyperplastic prostate tissues was constructed to analyze the correlations between *CXCL13* and clinical parameters. Results: *CXCL13* was highly expressed in the prostate tissues and upregulated in the BPH group. It was observed that *CXCL13* modulated cell proliferation, apoptosis, and the epithelial–mesenchymal transition (EMT) through CXCR5 via AKT and the ERK1/2 pathway in BPH-1, while it contributed to inflammation and fibrosis through CXCR5 via the STAT3 pathway in WPMY-1. In vivo, rHuCXCL13 induced the development of rat BPH. Additionally, *CXCL13* was positively correlated with the prostate volume and total prostate specific antigen. Conclusions: Our novel data demonstrated that *CXCL13* modulated cell proliferation, cell cycle, the EMT of epithelial cells, and induced the fibrosis of prostatic stromal cells via a variety of inflammatory factors, suggesting that *CXCL13* might be rediscovered as a potential therapeutic target for the treatment of BPH.

## 1. Introduction

Benign prostate hyperplasia (BPH) is a highly prevalent prostatic condition in elderly men [1], and the lower urinary tract symptoms (LUTS) caused by BPH are a bothersome complaint that has a major impact on the quality of life in the adult male population [1]. Aging and androgens are two well-established risk factors for the development and progression of BPH [1]. In the past several decades, many hypotheses about BPH have been proposed, including the imbalance of androgen–estrogen, the dysregulation of cell apoptosis and proliferation, the interaction of stromal and epithelial cells, the growth factor theory, the inflammation and cytokine theory, and genetic and familial factors [1]. However, the precise molecular etiology of this hyperplastic process still remains uncertain.

With the development of experimental technology, from PCR to next-generation high-throughput sequencing to multi-omics, more and more molecular mechanisms related to BPH will be elucidated. Among them, the identification of differentially expressed genes (DEGs) by transcriptome sequencing (RNA-seq) to explore the molecular mechanisms of disease, as well as the search for diagnostic markers and therapeutic targets, has become the direction of study many diseases, including BPH [2,3,4]. We and other groups performed RNA-seq on hyperplastic and normal prostate tissues and found a number of DEGs, among which C-X-C motif chemokine ligand 13 (*CXCL13*) was one of the most significantly upregulated DEGs with vague roles in BPH [3,4].

*CXCL13*, as a member of the CXC chemokine family, was also originally known as B-cell-attracting chemokine 1 (*BCA-1*) due to its functional characteristics. *BCA-1* could facilitate the specific chemotactic movement of B cells with a strong expression in the follicles of Peyer’s plaques, the spleen and lymph nodes. Several lines of evidence have indicated that *CXCL13* plays fundamental roles in inflammation, immune response [5,6,7,8] and fibrosis [8,9]. It has been documented that BPH is a type of inflammatory disease involving infiltration by a number of immune cells, including neutrophils and macrophages, etc. Chronic inflammation might directly stimulate the onset of BPH [10,11,12]. In clinical scenarios, BPH patients are usually complicated with prostatitis. Inflammatory infiltration could promote epithelial and stromal cells to secrete multiple key pro-inflammatory cytokines and inflammatory mediators [10,12]. In addition, recent data have suggested that both prostatic epithelial and stromal cells could express cytokine receptors and participate in the regulation of the local immune response as antigen-presenting cells [13,14]. Abundant experimental evidence has demonstrated that prostatic stromal and epithelial cells maintain a sophisticated paracrine type of communication. Isaacs, et al. revealed that stromal cell excretory protein partially regulated epithelial cell differentiation and proliferation [15], while studies of CXC motif chemokines such as *CXCL1*, *CXCL5*, *CXCL6* and *CXCL12* showed the complexity of the stromal–ECM–epithelial relationship. These chemokines were mainly expressed in the microenvironment, but were capable of stimulating the proliferation of both stromal and epithelial cells [1]. Indeed, it has been demonstrated that *CXCL13* could promote the proliferation of human mesenchymal stem cells, mesangial cells and osteoblasts [16,17,18]. Moreover, *CXCL13* is reported to be involved in cell apoptosis. *CXCL13* suppresses lymphocyte apoptosis in the spleen of pigs after porcine circovirus type 2 infection [19], while *CXCL13* inhibition induces the apoptosis of breast cancer cells [20]. In addition to cell proliferation, inflammation could cause a series of other chronic processes such as tissue damage, repair and remodeling [12,21]. Finally, chronic inflammation could lead to prostate fibrosis. Actually, various fibrotic diseases, such as liver, renal and cardiomyocyte fibrosis, could be attributed to inflammation. Fibrosis is characterized by the accumulation of stroma myofibroblasts, leading to the remodeling of the extracellular matrix and tissue hardening by secreting large amounts of collagen proteins deposited around it [22]. Importantly, the fibrosis of prostatic tissue around the urethra is an important pathological mechanism causing the dysfunction of the lower urinary tracts [23,24,25], which could contribute to disease progression, and the current first-line oral therapy (α-blockers and 5α-reductase inhibitors) have shown no effect on fibrosis. Additionally, several previous investigations have demonstrated that *CXCL13* is involved in the EMT process of breast cancer cells [26] and clear cell renal cell carcinoma cells [27]. However, the in-depth functional activities and mechanisms of this inflammatory protein in the development of BPH still remain unclear.

## 2. Results

### 2.1. The Expression and Localization of CXCL13 in Human Prostate Tissues and Cell Lines

**Figure 1:**
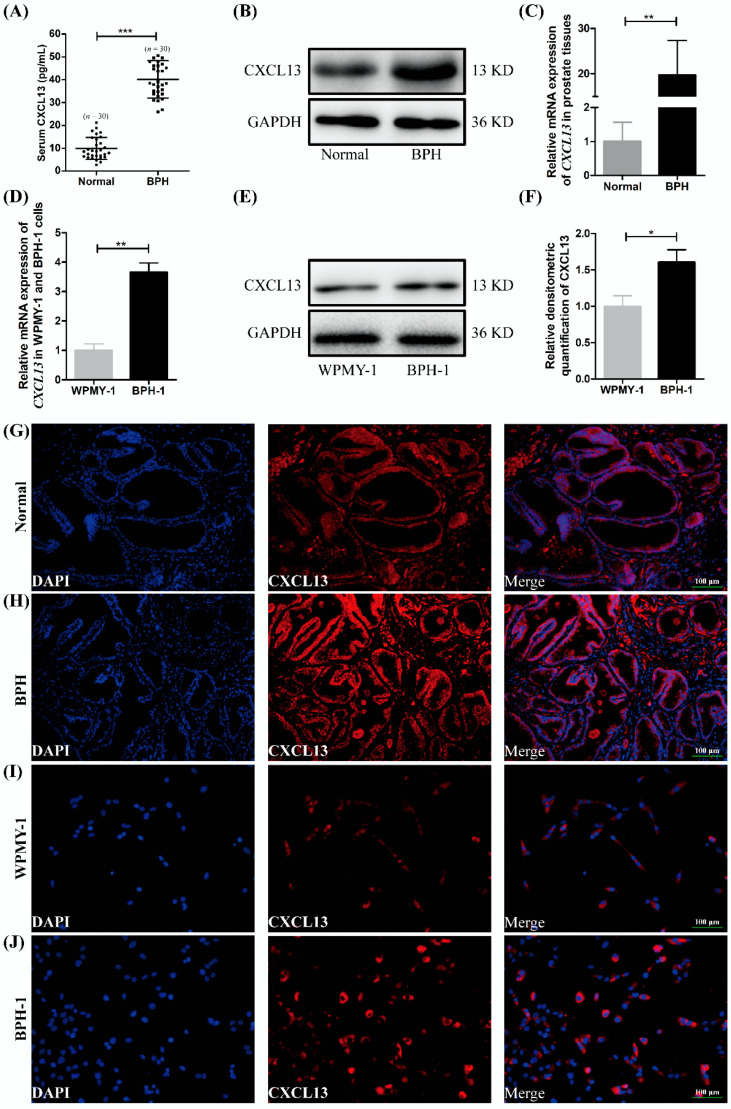
The expression of *CXCL13* in human serum, prostate tissue and cell lines. (**A**) Serum CXCL13 concentration in BPH patients and healthy controls. (**B**,**C**) The protein and mRNA expression of *CXCL13* in BPH tissues and normal ones. (**D**–**F**) The mRNA and protein expression of *CXCL13* in BPH-1 and WPMY-1 cells. Representative blots are shown. * *p* < 0.05, ** *p* < 0.01 and *** *p* < 0.001. (**G**,**H**) Immunofluorescence localization of CXCL13 in BPH tissues and normal ones. (**I**,**J**) Immunofluorescence localization of CXCL13 in BPH-1 and WPMY-1 cells. DAPI (blue) indicates nuclear staining and Cy3-immunofluorescence (red) indicates CXCL13 protein staining. Representative graphs are shown. All scale bars are 100 μm.

The serum concentration of CXCL13 in BPH patients was higher than that in healthy people (40.21 pg/mL versus 7.90 pg/mL, *p* < 0.001) (Figure 1A). Moreover, it was observed that the expression of *CXCL13* was increased at both the protein and mRNA levels in BPH samples when compared with normal ones (*p* < 0.01) (Figure 1B,C). We also detected the expression of *CXCL13* in cultured human BPH-1 and WPMY-1 cells and discovered higher expression of *CXCL13* at the mRNA and protein level in BPH-1 cells (Figure 1D–F). In addition, the immunofluorescence staining of prostate tissue demonstrated that CXCL13 was mainly localized in the epithelium of human prostate and was also expressed in the prostatic stroma (Figure 1G,H); positive and negative controls were not shown. Similarly, cell immunofluorescence staining showed that CXCL13 was expressed in both cells with more being stained in BPH-1 cells (Figure 1I,J).

### 2.2. rHuCXCL13 Treatment Promoted Proliferation and EMT of BPH-1 Cells, and Enhanced Inflammation and Fibrosis of WPMY-1 Cells

**Figure 2:**
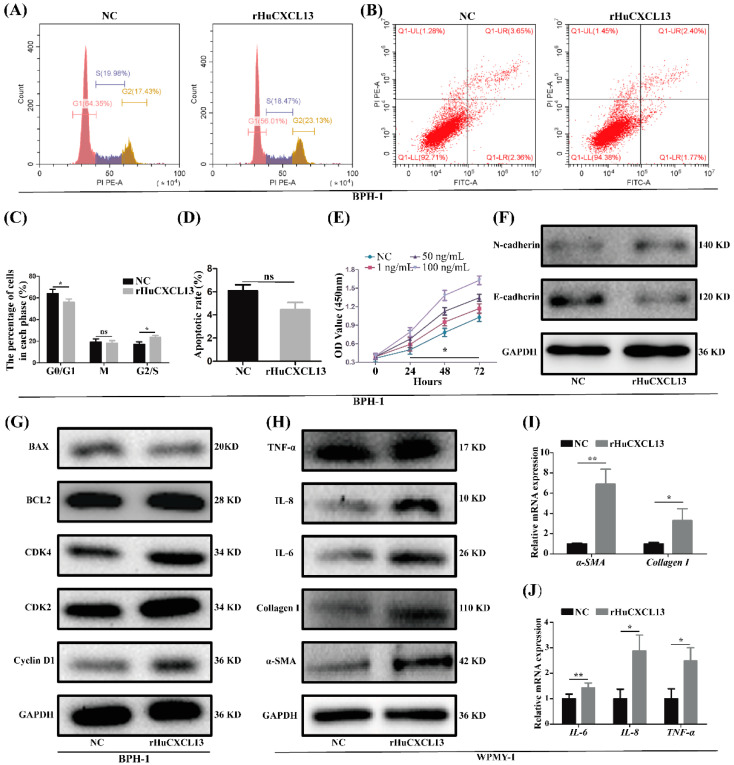
rHuCXCL13 treatment promoted proliferation of BPH-1 cells, fibrosis and inflammation of WPMY-1 cells. (**A**,**B**) Flow cytometry analysis of cell cycle and cell apoptosis in BPH-1 cells. (**C**,**D**) Statistical analysis of percentages (%) of cells at each stage and apoptotic rate (%) in BPH-1 cells. (**E**) CCK8 assay of BPH-1 cells. (**F**,**G**) Western blot assay of EMT-, cell-cycle- and apoptosis-related proteins in BPH-1 cells. (**H**–**J**) The mRNA and protein expression of markers of fibrosis and inflammation in WPMY-1 cells. * *p* < 0.05, ** *p* < 0.01 and ns means no significant difference.

To explore whether *CXCL13* could promote the proliferation of prostatic cells, BPH-1 and WPMY-1 cells were treated with 1, 50 and 100 ng/mL rHuCXCL13. The CCK8 assay demonstrated that exogenous rHuCXCL13 significantly enhanced the proliferation of BPH-1 cells in a dose-dependent manner (Figure 2E). Interestingly, the growth of WPMY-1 cells was not affected by rHuCXCL13 treatment (Figure S1A). In addition, the cell cycle and apoptosis of both cells treated with 100 ng/mL rHuCXCL13 were detected. Regarding the cell cycle, the G0/G1 phase of BPH-1 cells was reduced by approximately 8.13% (*p* < 0.05) (Figure 2A,C), while there were no obvious changes observed for WPMY-1 cells (*p* > 0.05) (Figure S1B,D). Moreover, the expression of proteins involved in G0/G1 phase regulation (Cyclin D1, CDK2, CDK4) were significantly increased in BPH-1 cells (Figure 2G), while unchanged in WPMY-1 cells (Figure S1F). As shown in Figure 2B,D and Figure S1C,E, the apoptotic rate was reduced by approximately 1.63% and 1.79% for BPH-1 and WPMY-1 cells, respectively. However, it did not reach statistical difference (*p* > 0.05) for either cell line. Similarly, proteins associated with apoptosis (BCL-2, BAX) showed no significant change (Figure 2G and Figure S1F). We also demonstrated that the addition of rHuCXCL13 could favor the EMT process of BPH-1 cells with E-cadherin decreased and N-cadherin increased (Figure 2F). Moreover, markers of fibrosis (*α-SMA*, *Collagen-I*) and inflammation (*TNF-α*, *IL-6*, *IL-8*) were obviously elevated at both the mRNA and protein level in WPMY-1 cells after rHuCXCL13 treatment (Figure 2H–J), while they were not detected in BPH-1 cells (data not shown).

### 2.3. Knockdown of CXCL13 Inhibited Proliferation, EMT and Promoted Apoptosis in BPH-1 Cells via ERK1/2 and AKT Pathway

**Figure 3:**
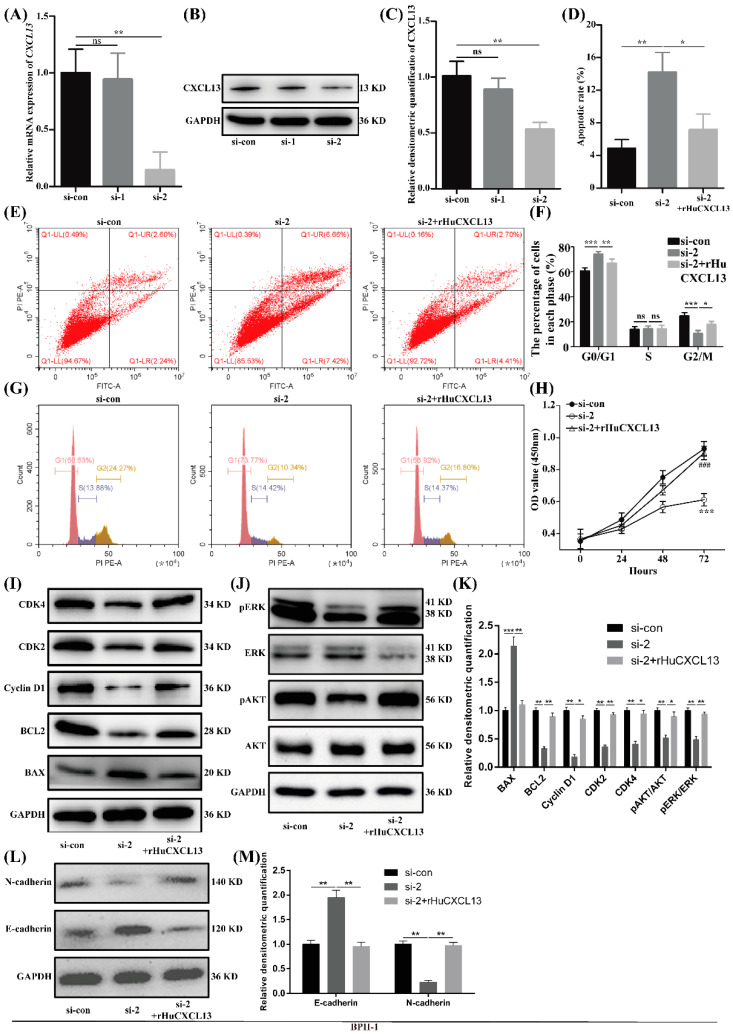
Knockdown of *CXCL13* inhibited proliferation, EMT and promoted apoptosis of BPH-1 cells via ERK1/2 and AKT pathway. (**A**–**C**) Knockdown efficiency of *CXCL13* at mRNA and protein levels. (**D**,**F**) Statistical analysis of apoptotic rate (%) and percentages (%) of cells at each stage. (**E**,**G**) Flow cytometry analysis of cell apoptosis and cell cycle. (**H**) CCK8 assay of BPH-1 cells. *** means *p* < 0.001 between si-con and si-2, ^###^ means *p* < 0.001 between si-2 and si-2+rHuCXCL13. (**I**,**J**) Western blot assay of cell-cycle-, cell-apoptosis- and pathway-related proteins. (**K**) Relative densitometric quantification of cell-cycle-, cell-apoptosis- and pathway-related proteins. (**L**) Western blot assay of EMT-related proteins. (**M**) Relative densitometric quantification of EMT-related proteins. * *p* < 0.05, ** *p* < 0.01, *** *p* < 0.001 and ns means no significant difference.

We further created a *CXCL13*-silenced model in BPH-1 cells. The knockdown efficiency of siRNA was validated by qRT-PCR and Western blot with si-2 reaching an 85% inhibitory effect (Figure 3A–C). Cell immunofluorescence staining also showed a significant downregulation of CXCL13 (Figure S2A). Therefore, si-2 was used in the subsequent experiments. Flow cytometry demonstrated that the knockdown of *CXCL13* could significantly amplify apoptosis (Figure 3D,E), make BPH-1 cells more stagnant at the G0/G1 phase (Figure 3F,G) and reduce proliferation activity (Figure 3H). Consistently, the Western blot study found that the pro-apoptotic protein BAX was increased and the anti-apoptotic protein BCL-2 was significantly decreased when *CXCL13* was silenced (Figure 3I,K). Meanwhile, the proteins involved in the regulation of the G0/G1 phase (Cyclin D1, CDK2, CDK4) showed a significant decrease (Figure 3I,K). Moreover, the protein levels of pAKT and pERK1/2 were significantly lowered with AKT and ERK1/2 being unchanged following *CXCL13* knockdown (Figure 3J,K). In addition, the knockdown of *CXCL13* inhibited the EMT process with E-cadherin enlarged and N-cadherin attenuated (Figure 3L,M). Nevertheless, the effects of *CXCL13* knockdown on the cell proliferation, apoptosis and EMT progress of BPH-1 cells were almost completely reversed with 100 ng/mL rHuCXCL13 treatment (Figure 3).

### 2.4. Overexpression of CXCL13 Upregulated Inflammation and Fibrosis in WPMY-1 Cells through STAT3 Pathway

**Figure 4:**
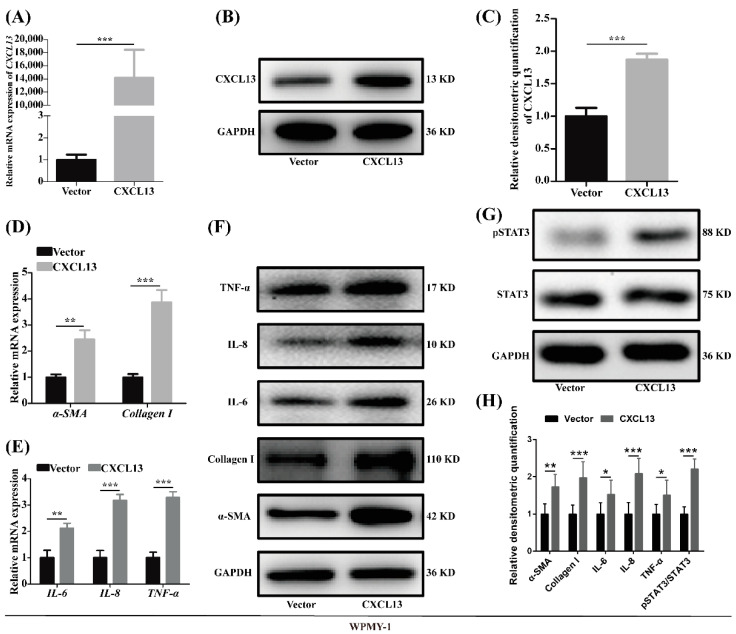
Overexpression of *CXCL13* promoted inflammation and fibrosis of WPMY-1 cells through STAT3 pathway. (**A**–**C**) Overexpression efficiency of *CXCL13* at the mRNA and protein levels. (**D**–**F**) The mRNA and protein level of fibrosis and inflammation markers after *CXCL13* overexpression. (**G**) The protein level of STAT3 pathway after *CXCL13* overexpression. (**H**) Relative densitometric quantification of fibrosis-, inflammation- and pathway-related proteins. * *p* < 0.05, ** *p* < 0.01 and *** *p* < 0.001.

Given that the expression of *CXCL13* was relatively low in WPMY-1 cells, the *CXCL13* plasmid was transfected to overexpress *CXCL13*. The overexpression efficiency was confirmed with qRT-PCR, Western blot and immunofluorescence (Figure 4A–C and Figure S2B). It is interesting that fibrosis markers (*α-SMA*, *collagen I*) and inflammation markers (*IL-6*, *IL-8*, *TNF-α*) were all more expressed both at the mRNA and protein level after the overexpression of *CXCL13* in WPMY-1 cells (Figure 4D–F,H). Moreover, the protein level of pSTAT3 was significantly heightened with STAT3 unchanged following plasmid transfection (Figure 4G,H). Consistent with rHuCXCL13 treatment, the overexpression of *CXCL13* also showed no effect on cell proliferation or apoptosis in WPMY-1 cells (data not shown).

### 2.5. Anti-CXCR5 Could Rescue the Alternations Induced by rHuCXCL13

**Figure 5:**
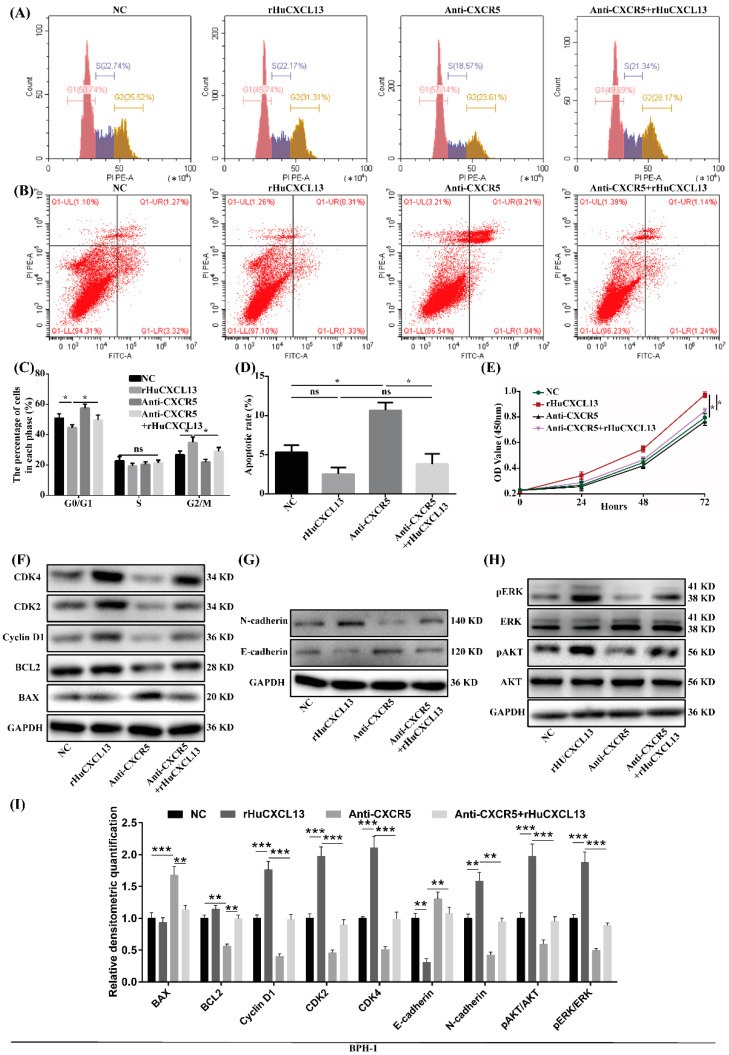
Anti-CXCR5 (1 μg/mL) could rescue the alternations induced by rHuCXCL13 in BPH-1 cells. (**A**,**B**) Flow cytometry analysis of cell cycle and cell apoptosis. (**C**,**D**) Statistical analysis of percentages (%) of cells at each stage and apoptotic rate (%). (**E**) CCK8 assay. (**F**–**H**) Western blot assay of cell-cycle-, apoptosis-, EMT- and pathway-related proteins. (**I**) Relative densitometric quantification of cell-cycle-, apoptosis-, EMT- and pathway-related proteins. * *p* < 0.05, ** *p* < 0.01, *** *p* < 0.001 and ns means no significant difference.

**Figure 6:**
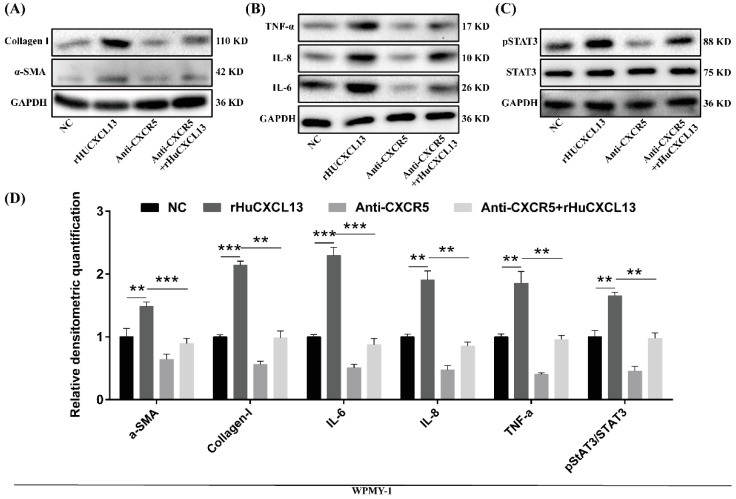
Anti-CXCR5 (1 μg/mL) could rescue the alternations induced by rHuCXCL13 in WPMY-1 cells. (**A**–**C**) Western blot assay of fibrosis-, inflammation-, and pathway-related proteins. (**D**) Relative densitometric quantification of fibrosis-, inflammation-, and pathway-related proteins. ** *p* < 0.01 and *** *p* < 0.001.

We further utilized anti-CXCR5 to verify the potential mechanism of *CXCL13*. As previously mentioned, the addition of rHuCXCL13 could promote the transition from the G0/G1 phase to the G2/M phase, enhance cell proliferation and facilitate the EMT process in BPH-1 cells, while the addition of anti-CXCR5 partially blocked these processes (Figure 5). Similarly, anti-CXCR5 also rescued the alternations of fibrosis and inflammation induced by rHuCXCL13 in WPMY-1 cells (Figure 6).

### 2.6. rHuCXCL13 Induces the Development of BPH In Vivo

**Figure 7:**
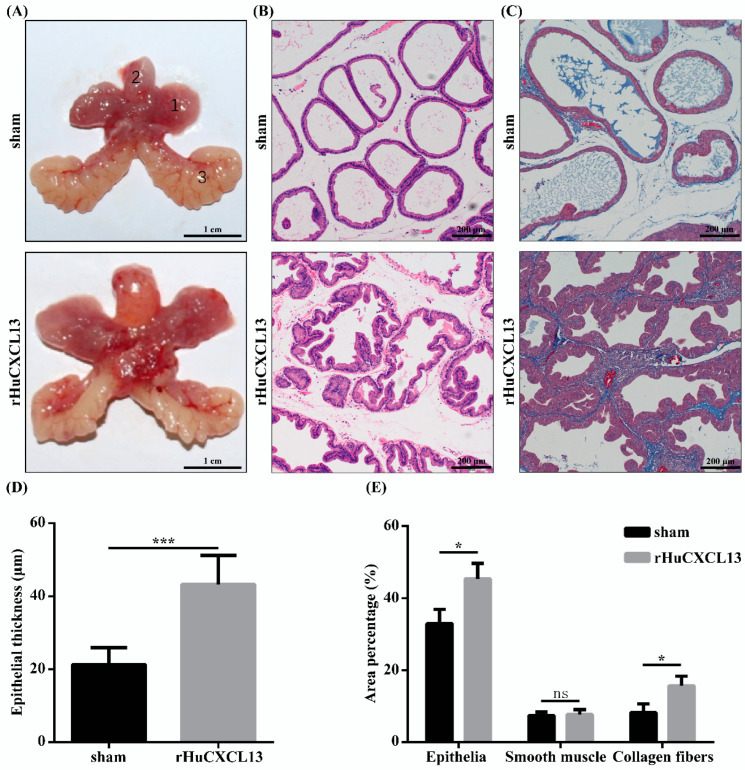
rHuCXCL13 induces the development of BPH in vivo. (**A**) The rat urogenital tissues from sham and rHuCXCL13-treated rats. (1) Ventral prostate, (2) bladder and (3) seminal vesicle. (**B**) Representative H&E staining of sham and rHuCXCL13-treated rat prostates. (**C**) Masson’s trichrome staining of sham and rHuCXCL13-treated rat prostates. Prostate epithelial cells were stained orange, SM cells were stained red, and collagen fibers were stained blue. (**D**) Statistical analysis of epithelial thickness of sham and rHuCXCL13-treated rat prostates. (**E**) Quantification of Masson’s trichrome staining. The scale bars are 200 μm. * *p* < 0.05, *** *p* < 0.001 and ns means no significant difference.

**Figure 8:**
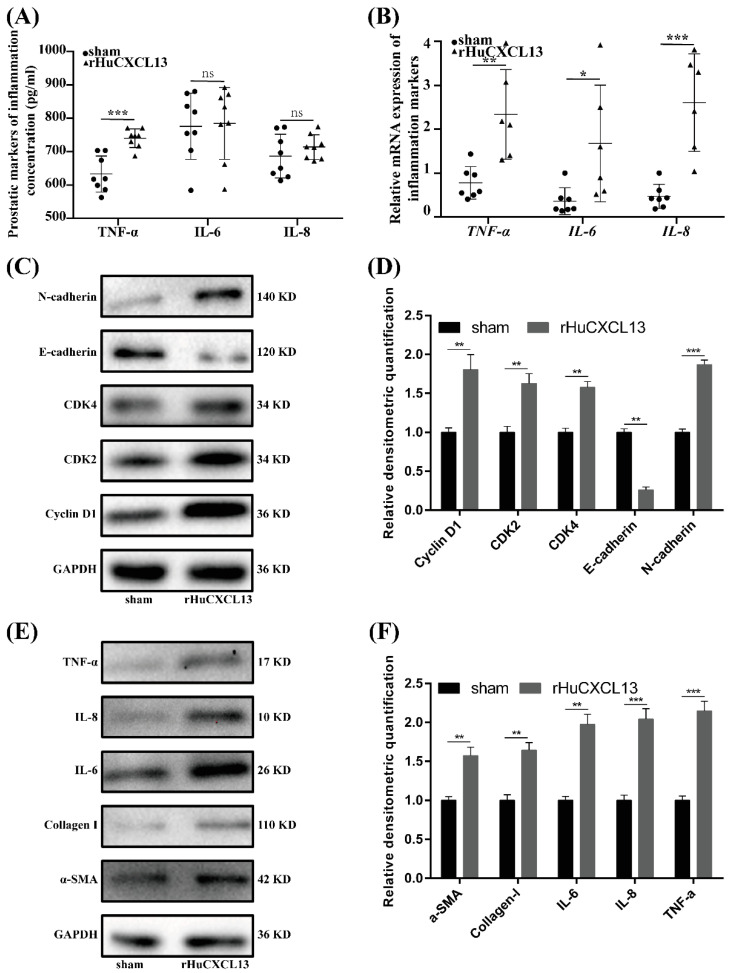
rHuCXCL13 induces the development of BPH in vivo. (**A**) Concentration of inflammation markers in rat blood. (**B**) Relative mRNA expression of inflammation markers in rat prostate tissues. (**C**) Western blot assay of cell-cycle- and EMT-related proteins in sham and rHuCXCL13-treated rat prostate tissues. (**D**) Relative densitometric quantification of cell-cycle- and EMT-related proteins in sham and rHuCXCL13-treated rat prostate tissues. (**E**) Western blot assay of fibrosis- and inflammation-related proteins in sham and rHuCXCL13-treated rat prostate tissues. (**F**) Relative densitometric quantification of fibrosis- and inflammation-related proteins in sham and rHuCXCL13-treated rat prostate tissues. * *p* < 0.05, ** *p* < 0.01, *** *p* < 0.001 and ns means no significant difference.

Finally, we translated our in vitro study into in vivo experiment by injecting rHuCXCL13 into the ventral prostate of rats. As shown in Figure 7A and Table 1, the weight of the ventral prostate was obviously increased after four weeks of treatment with rHuCXCL13. Additionally, prostate index (prostate wet weight (mg)/body weight (g)) was also upregulated in the rHuCXCL13-treated rats (Table 1). Histologically, the epithelial component was relatively increased and the glands were protruded into the lumen (Figure 7B). Moreover, the average thickness of epithelium was significantly augmented after rHuCXCL13 treatment (Figure 7D). Masson’s trichrome staining further demonstrated that the hyperplasia of the prostate occurred mainly in the epithelium and collagen fibers, whereas smooth muscle showed no difference in BPH rats (Figure 7C,E). Similar to the in vitro study, all inflammation markers were upregulated in the rat prostate. However, only TNF-α was increased in the rat serum (Figure 8A,B). Then, we detected all the aforementioned proteins associated with cell cycle, EMT, fibrosis and inflammation in the rat model, whose alternations were consistent with the cell models (Figure 8C–F).

### 2.7. TMA Showed the Correlations of CXCL13 with Clinical Parameters

**Figure 9:**
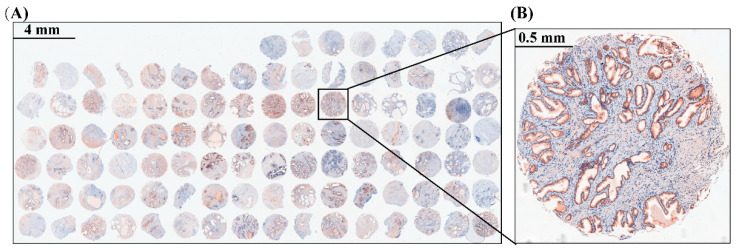
Immunohistochemical of CXCL13 on TMA of BPH. (**A**) A holistic view of CXCL13 stained TMA. The scale bar is 4 mm. (**B**) Representative graph of CXCL13 stained TMA. The scale bar is 0.5 mm.

Additionally, we collected 104 benign hyperplastic prostate tissues and performed a TMA (Figure 9). Descriptive statistics of these patients’ clinical parameters are listed in Table 2. Further analysis of the TMA demonstrated that *CXCL13* expression was positively correlated with PV and tPSA, while no association was observed for other clinical parameters (Table 3).

## 3. Discussion

Our novel data demonstrated that *CXCL13* was mainly localized in the epithelium with less expression in the stroma of human prostate tissues, and it was upregulated in the prostate tissues and serum from BPH patients. We also showed that *CXCL13* could regulate the imbalance between cell apoptosis and cell proliferation, as well as modulate cell cycle and EMT processes through CXCR5 via the AKT and ERK1/2 pathways in epithelial cells, while it could promote fibrosis and inflammation processes through CXCR5 via the STAT3 pathway in the stroma.

In our current study, *CXCL13* was observed to be abundantly expressed in human prostate tissues. We further demonstrated that the CXCL13 protein was mainly localized in epithelia while less observed in stroma of the human prostate, which was consistent with a previous investigation [28]. Moreover, *CXCL13* was found to be upregulated both in the prostates and serum from BPH patients (*n* = 30). Similarly, ELISA performed by Singh, et al. showed that the serum CXCL13 level was higher in BPH patients (*n* = 10) when compared with normal healthy donors (*n* = 10) [29].

In recent decades, dozens of inflammatory pathways have been found to contribute to BPH pathogenesis. As a chemokine and one of the most differentially expressed genes in the hyperplastic prostate, the current study showed that *CXCL13* plays important roles in the BPH process. Firstly, we demonstrated that elevated *CXCL13* could enhance the proliferation of the epithelial cell line BPH-1, which was similar to several previous studies. Li, et al. discovered that *CXCL13* could enhance the proliferation of human mesenchymal stem cells through the MAPK pathway [16], while Da, et al. found that *CXCL13* enhanced mesangial cell growth via combination with CXCR5 in SLE [17]. Additionally, *CXCL13* could induce the proliferation of osteoblasts in osteoarthritis patients [18]. Additionally, we observed that the addition of CXCL13 could increase the transition from the G0/G1 phase to the G2/M phase, which was in line with previous studies [17,30]. However, it had no effect on the modulation of the cell cycle or the growth of the stromal cell line WPMY-1. Consistently, Middleton, et al. observed that the addition of rHuCXCL13 did not affect the cell proliferation of WPMY-1 [3]. The differential expression of CXCR5 could be attributed to this discrepancy. Indeed, our study demonstrated that CXCR5 was more expressed in BPH-1 cells than in WPMY-1 cells. Moreover, *CXCL13* could mediate the apoptosis of prostate cells. In our current study, *CXCL13* knockdown could trigger cell apoptosis but *CXCL13* overexpression showed no effect on cell death. As the apoptosis level in the control cells was very low, rHuCXCL13 could not further attenuate apoptosis. Several previous studies have also revealed that *CXCL13* suppresses lymphocyte apoptosis in the spleen of pigs after porcine circovirus type 2 infection [19], while *CXCL13* inhibition induces the apoptosis of breast cancer cells through blocking the CXCR5/ERK pathway [20]. In addition to the imbalance of cell proliferation and apoptosis, *CXCL13* also favored the EMT process, which is involved in BPH development [31,32,33]. It represents the accumulation of mesenchymal-like cells derived from the prostatic epithelium [34,35,36,37]. The present study observed that exogenous rHuCXCL13 amplified the EMT with E-cadherin decreased while N-cadherin increased. On the other hand, silenced *CXCL13* or anti-CXCR5 reversed the rHuCXCL13-induced EMT. Similarly, CXCL13-CXCR5 co-expression regulated the EMT of breast cancer cells during lymph node metastasis [26]; additionally, CXCL13 secreted by M2 macrophages could promote the EMT of clear cell renal cell carcinoma [27]. We further found that the protein level of pAKT and pERK1/2 was significantly lowered with *CXCL13* knockdown, which was consistent with previous reports [17,38,39]. Therefore, we speculated that *CXCL13* may participate in the development of BPH through modulating the balance between cell proliferation and cell apoptosis, as well as EMT, via AKT and ERK1/2 pathways.

Although both *CXCL13* and its receptor CXCR5 were less expressed in the stromal WPMY-1 cells, it is interesting that the fibrosis markers (*α-SMA*, *collagen I*) and inflammation markers (*IL-6*, *IL-8*, *TNF-α*) were all amplified when *CXCL13* was overexpressed. However, *CXCL13* showed no inflammatory effect on BPH-1 cells (data not shown), which is required to be further investigated. Actually, *CXCL13* was found to be positively correlated with IL-6 in prostate cancer, BPH and high-grade prostatic intraepithelial neoplasia [29], and involved in experimental autoimmune cystitis and interstitial cystitis [40]. Moreover, *CXCL13* could be a biomarker of inflammation in a diversity of diseases, including multiple sclerosis, neuromyelitis optica and rheumatoid synovium [41,42]. Importantly, inflammation not only played instrumental roles in cell proliferation but also in the formation of fibrosis. In fact, fibrosis can be thought of as an inflammatory-reaction-induced abnormal wound-healing progress [43]. Vuga, et al. revealed that *CXCL13* was a prognostic biomarker in patients with idiopathic pulmonary fibrosis [44]. Recent studies have suggested that fibrosis may act as a pathological factor that contributes to BPH/LUTS [23,45,46,47]. Anti-fibrosis therapy is considered as a potential effective approach for BPH patients, especially for those who failed to respond to the first-line drugs, including α1-adrenergic receptor agonists and 5α-reductase inhibitors [24]. Moreover, the protein level of pSTAT3 was significantly heightened with STAT3 unchanged following plasmid transfection, indicating that *CXCL13* mediated fibrosis via STAT3 signaling.

We further translated our in vitro studies to in vivo. Compared with the sham group, the weight of the ventral prostate was obviously increased in the rHuCXCL13-treated rats. The histology analysis demonstrated that collagen fibers, the epithelial component, were relatively increased and glands were protruded into the lumen, which are features of hyperplasia. Additionally, all the inflammation markers were upregulated in the rat prostate, while only TNF-α was increased in the rat serum. We speculated that it could be related to the intraprostatic injection of rHuCXCL13, instead of systemic delivery. In addition, markers of the cell cycle, EMT, inflammation and fibrosis were consistently altered at the cellular level. In short, it strongly suggested that *CXCL13* promoted the development of BPH in vivo.

Finally, we found that the expression of *CXCL13* was positively correlated with PV and tPSA, with no relation to other parameters, including age, body mass index (BMI), free PSA (fPSA), the maximum flow rate (Qmax), residual urine (RUV), international prostate symptom score (IPSS) or nocturnal enuresis. Singh, et al. also discovered that serum *CXCL13* levels were significantly (*p* < 0.0001) correlated with serum tPSA [29]. PSA is a marker for prostate cancer and BPH [1,48], and several earlier studies consistently indicated a positive correlation between PSA and PV [49,50,51]. The non-correlation of *CXCL13* with IPSS will need further validation. Coincidentally, Singh, et al. discovered that there was no overlap between serum CXCL13 concentrations in prostate cancer patients, BPH patients and healthy donors [29]. However, this distinction could not be made when PSA was used as a predictive factor of prostatic disease. Therefore, we speculated that CXCL13 may be a better predictor of prostatic diseases than PSA. Of course, this still needs to be further verified with large samples.

Collectively, our current study revealed that *CXCL13* was mainly localized in the epithelial compartment of human prostate tissues and it was upregulated in prostate tissues and serum from BPH patients. The knockdown of *CXCL13* induced cell apoptosis, cell cycle arrest at the G0/G1 phase, and inhibited cell proliferation and the EMT process in BPH-1 cells through CXCR5 via the AKT and ERK1/2 pathways, while the overexpression of *CXCL13* or the addition of rHuCXCL13 exhibited a promotion of prostate inflammation and the fibrosis process in WPMY-1 cells, via the STAT3 pathway. Our novel data suggest that *CXCL13* plays multiple vital roles in the development of BPH, and it could be rediscovered as a new therapeutic target to ablate the initial and development of BPH. However, an intriguing model to explore the in vivo function of *CXCL13* utilized by genetic mice with the conditional knockout of the *CXCL13* gene in the prostate is in progress.

## 4. Materials and Methods

### 4.1. Animals, Tissues and Serum

A total of 16 specific-pathogen-free (SPF) grade male Sprague–Dawley rats (6 weeks old) weighing 200–250 g was used and randomly divided into two groups (*n* = 8 per group): the sham group and rHuCXCL13 group. All surgical procedures were performed under anesthesia by intraperitoneal injection of 35 mg/kg pentobarbital sodium (Abbott Laboratory, Chicago, IL, USA). A stock solution of rHuCXCL13 (Cat. 10621-HNAE, Sino Biology lnc., Beijing, China) was made in sterile normal saline. Rats underwent small midline incisions of the lower abdomen above the penis, and the ventral prostates were exposed. With a 30-gauge needle, 0.5 ng of rHuCXCL13 in a final volume of 50 μL sterile normal saline were injected into both the right and left ventral lobes of the prostate. For the sham group, 50 μL sterile normal saline were injected. After the injection, 2% lidocaine solution was applied to the wound, and then the wound was closed. After 4 weeks of post-surgery, rats were euthanized; the ventral prostate, bladder and seminal vesicle were harvested and weighed. Prostate tissues were divided into two strips and were, respectively, stored in liquid nitrogen for PCR and Western blotting analysis, and stored in 10% neutral buffered formalin for histological examination. Additionally, 2 mL blood were collected to obtain serum for ELISA. Animal experiments were conducted at the Animal Center of Zhongnan Hospital, Wuhan University and all animal protocols were approved by the Medical Ethics Committee for Experimental Animals of Zhongnan Hospital, Wuhan University.

Normal prostate tissue was obtained from 8 young brain-dead men (28.2 ± 4.4 years old) undergoing organ donation at the Organ Transplant Center of Zhongnan Hospital, with pathological examination revealing no hyperplasia. A total of 104 specimens of BPH samples (70.0 ± 7.5 years old) with clinical parameters were obtained from the patients who underwent transurethral resection prostate in Department of Urology, Zhongnan Hospital of Wuhan University, and 30 swatches of serum of BPH patients (70.1 ± 6.9 years old) were gathered incidentally. The serum of healthy volunteers (26.7 ± 3.2 years old) was collected from the Physical Examination Center in the same hospital. All volunteers were males and their age ranged from 20–35 years old. Prostate ultrasound showed a normal-sized prostate. Volunteers with allergies, asthma or associated inflammatory diseases were excluded. Prostate tissues were divided into two strips and were, respectively, stored in liquid nitrogen for PCR and Western blotting analysis, and stored in 10% neutral buffered formalin for immunofluorescence microscopy and the construction of TMA. All human samples were obtained after the approval of the Hospital Committee for Investigation in Humans and after receiving written informed consent from all patients or their relatives. All human studies were conducted in accordance with the principles of the Declaration of Helsinki.

### 4.2. TMA Construction and Immunohistochemistry Analysis

A total of 104 paraffin-embedded specimens of BPH samples were sliced followed by H&E staining. Then, the representative areas of these sections were evaluated and confirmed by a senior pathologist. One core (1.5 mm diameter) was removed from each paraffin-embedded sample and inserted into a blank paraffin block. The resultant TMA was cut into 4 μm sections, which were mounted on microscope slides. In short, the paraffin section was deparaffinized first, then antigen retrieval was performed in citric acid buffer (pH 6.0), and endogenous peroxidase activity was blocked in 0.3% H_2_O_2_. Subsequently, the slide was incubated with primary and then secondary antibody prior to visualization by peroxidase and 3,3′-diaminobenzidine tetrahydrochloride. The stained section was imaged using an Olympus-DP72 light microscope (Olympus, Tokyo, Japan). The staining intensity was quantified by two pathologists in a blinded manner. Then, the percentage of the target-protein-positive area was measured by Image J [52].

### 4.3. Cell Culture

Human benign prostatic enlargement epithelia cell line BPH-1 (Cat. BNCC339850) was purchased from the Procell Co., Ltd. in Wuhan, China. Identification of the cell lines was performed at the China Center for Type Culture Collection in Wuhan, China. SV40 large-T antigen-immortalized stromal cell line WPMY-1 (Cat. GNHu36) was purchased from the Stem Cell Bank, Chinese Academy of Sciences in Shanghai, China. The BPH-1 cells were cultured in RPMI-1640 medium (Gibco, Grand Island, NY, USA) containing 10% fetal bovine serum (FBS) (Gibco, Grand Island, USA). The WPMY-1 cells were cultured in DMEM medium (Gibco, Grand Island, USA) containing 1% penicillin G sodium/streptomycin sulfate and 5% FBS. All the cell lines were recently authenticated and were cultured in a humidified atmosphere consisting of 95% air and 5% CO_2_ at 37 °C.

### 4.4. Addition of rHuCXCL13 in BPH-1 and WPMY-1 Cells

BPH-1 and WPMY-1 cells were seeded in a 6-well plate and incubated to 30% confluence. Then, the experimental group was treated with 100 ng/mL rHuCXCL13 and the control group was cultured with the same amount of DEPC water for 48 h.

### 4.5. Addition of Anti-CXCR5 in BPH-1 and WPMY-1 Cells

BPH-1 and WPMY-1 cells were seeded in a 6-well plate and incubated to 30% confluence. Then, all cells were divided into four groups: NC group (DEPC water), rHuCXCL13 group (100 ng/mL rHuCXCL13), anti-CXCR5 group (1 μg/mL anti-CXCR5) and rHuCXCL13 + anti-CXCR5 group (100 ng/mL rHuCXCL13 + 1 μg/mL anti-CXCR5). Cells were harvested for further investigation after 48 h.

### 4.6. Knockdown of CXCL13 in BPH-1 Cells

BPH-1 cells were seeded in a 6-well plate and incubated to 40–60% confluence. According to the manufacturer’s instructions, 100 pmoL siRNA (Genepharma, Suzhou, China) and 5 μL Lipofectamine^®^2000 (Invitrogen, Carlsbad, CA, USA) were added to two tubes of 200 μL serum-free medium, respectively, and then both were blended and kept still for 20 min. After changing 1.6 mL fresh RPMI-1640 medium in each well, the mixtures were added. BPH-1 cells were transfected with *si-CXCL13* for 4–6 h, and then incubated with fresh RPMI-1640 medium for 48–72 h. Transfection efficiency was determined by qRT-PCR, Western blot and immunofluorescence staining. The sense sequences of *si-CXCL13* are listed in Table S1.

### 4.7. Overexpression of CXCL13 in WPMY-1 Cells

The *CXCL13* plasmid was synthesized by Fenghui biology Co., Ltd. in Changsha, China. WPMY-1 cells were inoculated in a 6-well plate and incubated to up to 50–70% confluence. According to the manufacturer’s instructions, 2 μg *CXCL13* cDNA and 5 μL Lipofectamine^®^2000 (Invitrogen, Carlsbad, USA) were added to serum-free medium in 200 μL, respectively, and then both were blended for 20 min. After changing 1.6 mL fresh DMEM medium in each well, the mixtures were added. WPMY-1 cells were transfected with *CXCL13* cDNA for 4–6 h, and then incubated with fresh DMEM medium for 48–72 h. Transfection efficiency was determined by qRT-PCR, Western blot and immunofluorescence staining.

### 4.8. Total RNA Extraction, RNA Reverse Transcription and qRT-PCR Analysis

Total RNA was extracted by Hipure Total RNA Mini Kit (Cat. R4111-03, Magen, Guangzhou, China) according to the manufacturer’s instructions, and quantified at 260/280 nm with Nanophotometer spectrophotometer (Thermo Fisher Scientific, Shanghai, China). The cDNA was synthesized using 1 μg total RNA by ABScript II RT Master Mix for qPCR (Cat.RK20402, Abclonal, Wuhan, China). Each qPCR reaction was conducted with 10 μL 2X Universal SYBR Green Fast qPCR Mix (Cat.RK21203, Abclonal, Wuhan, China), 7 μL ddH_2_O, 1 μL cDNA, 1 μL forward primer and 1 μL reverse primer. The expression levels of genes were normalized to the expression of *GAPDH* mRNA and compared by the 2^−ΔΔCT^ method. All samples were run in triplicate. Primer sequences are listed in Table 4.

### 4.9. Western Blotting Analysis

Tissues and cells were lysed and ultrasonicated in RIPA reagent containing protease inhibitor and phosphatase inhibitor (Sigma-Aldrich, St. Louis, MO, USA) on ice for 30 min. After centrifugation at 12,000× *g* for 15 min at 4 °C, the supernatant was collected. Then, protein concentration was measured by bicinchoninic acid (BCA) assay. Protein was separated by sodium dodecyl sulfate–polyacrylamide gel (SDS–PAGE) and transferred to polyvinylidene fluoride membrane (PVDF, Millipore, Billerica, MA, USA) using a Bio-Rad wet transfer system. The membrane was then blocked in tris-buffered saline with 0.05% tween 20 (TBST) buffer (pH 6.0) containing 5% non-fat dry milk, and incubated sequentially with primary and secondary antibodies (listed in Table 5 and Table 6). An enhanced chemiluminescence kit (Thermo Fisher Scientific, Shanghai, China) was used to expose the bands. The expression level of proteins was normalized to the expression of GAPDH. All samples were independently repeated three times and the means were determined.

### 4.10. Flow Cytometry Analysis

A total of 1 × 10^6^ cells was collected from the two types of cells cultured by aforementioned method. The apoptosis rate was detected by flow cytometry (Cat. FC500, Beckman Coulter) using the cell apoptosis kit (Cat. AP101) provided by Multisciences Biotech, CO., Ltd., Hangzhou, China. According to the instructions of the cell cycle detection kit (Cat. CCS012) provided by the same manufacturer, the DNA content distribution was analyzed by flow cytometry to detect the cell cycle.

### 4.11. Cell Counting Kit-8 (CCK8) Assay

After transfection or addition for 48 h, 3000–5000 cells/200 μL medium were seeded in 96-well plates to grow for another three days. Exactly 10 µL CCK8/100 μL medium was added to each well on day 0, 1, 2 and 3, and the cells were incubated for 2 h at 37 °C in an incubator. Finally, we measured the absorbance at 450 nm with a microplate reader (Thermo Labsystems, Vantaa, Finland).

### 4.12. Immunofluorescent Staining for Tissues

Tissues were sectioned in 10 μm thick slices and thawed, mounted onto glass slides using a cryostat (Leica CM 1850, Wetzlar, Germany), air-dried, and fixed for 10 min in ice-cold acetone. Slides were washed in PBS and incubated for 2 h in a mixture of PBS supplemented with 0.2% Triton X-100 and 0.1% bovine serum albumin, followed by incubation overnight with the primary antibodies (listed in Table 5). The secondary antibody (listed in Table 6) employed to visualize the localization of CXCL13 was Cy3-conjugated goat anti-rabbit IgG (1:1000). DAPI was used for staining the nucleus. Visualization was performed with a Laser Scanning Confocal Microscope (Olympus, Tokyo, Japan).

### 4.13. Immunofluorescent Staining for Cells

For cell immunofluorescence microscopy, cells were cultured as aforementioned, followed by seeding on 12 mm coverslips and washing by ice-cold phosphate-buffered saline (PBS, pH = 7.4). The coverslips were then fixed with 4% paraformaldehyde (PFA) for 30 min, followed by 0.1% Triton X-100 incubation and blocking in goat serum for 30 min at room temperature. Then, they were incubated with primary antibody (listed in Table 5) at room temperature for 2 h, washed with PBS and incubated with Cy3-labeled or FITC-labeled secondary antibody (listed in Table 6) for 1 h. Nuclei were labeled with DAPI (2 μg/mL). Visualization was performed with a Laser Scanning Confocal Microscope (Olympus, Tokyo, Japan).

### 4.14. Hematoxylin and Eosin (H&E) Staining

Prostate paraffin sections (5 μm) were deparaffinized in xylene for 3 × 10 min, then rehydrated in descending concentrations of ethanol (100%, 96%, 80%, 70%) and H_2_O. The sections were then stained in 10% Hematoxylin (Sigma-Aldrich, St. Louis, USA) for 7 min, followed by washing under tap water for 10 min to reveal the nuclei. Afterwards, the sections were stained in 1% Eosin (Sigma-Aldrich, St. Louis, USA) containing 0.2% glacial acetic acid for 5 min. After staining, the sections were washed with tap water, dehydrated in increasing grades of ethanol (70%, 80%, 96%, 100%), and cleared in xylene for 3 × 10 min. The sections were imaged by an Olympus-DP72 light microscope (Olympus, Tokyo, Japan).

### 4.15. Masson’s Trichrome Staining

Prostate tissues were embedded into paraffin after being fixed in 10% formalin for 24–36 h and cut into 5 μm sections. Then, the sections were stained using Masson’s trichrome staining. Staining was detected by light microscopy. Prostatic smooth muscle (SM) cells, collagen fibers, and epithelial cells were stained red, blue, and orange, respectively. The area percentages of SM, collagen fibers, and glandular epithelium were quantitated with Image Pro Plus 5.0.

### 4.16. Enzyme-Linked Immune Sorbent Assay

According to the manufacturer’s instructions, human CXCL13 ELISA kit (Abcam, Cambridge, UK, Cat. ab179881) was used to detect serum CXCL13 concentration in BPH patients and healthy controls. Rat IL-6, IL-8 and TNF-α ELISA kit (Jiangsu Meimian industrial Co., Ltd, Yancheng, China. Cat. MM-0190R2, MM-0175R2, MM-0180R2) were used to detect the concentration of IL-6, IL-8 and TNF-α in rat serum.

### 4.17. Statistical Analysis

Statistical analyses were performed using SPSS statistical software, version 21.0 (IBM, Chicago, IL, USA). The data are expressed as means ± standard deviations from at least three independent experiments. Student’s two-tailed *t*-test was used to compare the means of two-group samples, and one-way ANOVA was applied for the comparison of multiple groups. *p* < 0.05 was considered statistically significant.

## Figures and Tables

**Table 1:** Variation of biometric and physiological parameters in sham and rHuCXCL13-treated rats.

Group	Body Weight (g)		Ventral Prostate Weight (mg)	Prostate Index
	Initial	Final		
sham	337.6 (17.9)	473.2 (37.8)	598.7 (81.1)	1.3(0.3)
rHuCXCL13	339.1 (8.1)	468.3 (38.2)	759.8 (83.1) *	1.7 (0.2) *

* *p* < 0.05.

**Table 2:** The descriptive statistics of clinical parameters of BPH patients.

	Mean	SD
CXCL13 score	0.6	0.4
Age (years)	70.1	7.5
BMI (kg/m^2^)	22.8	2.8
PV (cm^3^)	60.8	36.7
tPSA (ng/mL)	7.0	5.9
fPSA (ng/mL)	1.6	1.4
Qmax (mL/s)	10.0	5.9
RUV (mL)	160.7	114.5
IPSS	21.7	7.4
nocturnal enuresis	3.1	2.0

BMI: body mass index; PV, prostate volume; tPSA, total prostate-specific antigen; fPSA, free prostate-specific antigen; Qmax, maximum flow rate; RUV, residual urine volume; IPSS, international prostate symptom score.

**Table 3:** The correlations between clinical parameters and *CXCL13* expression of BPH patients.

	Correlation Coefficient	*p* Value
Age	−0.150	0.131
BMI	0.124	0.211
PV	0.230	0.028 *
tPSA	0.235	0.027 *
fPSA	0.149	0.167
Qmax	0.112	0.502
RUV	-0.060	0.687
IPSS	0.074	0.457
nocturnal enuresis	0.040	0.687

* *p* < 0.05.

**Table 4:** Primer sequence used for qPCR.

Target Gene		Human (5′ to 3′)
*CXCL13*	Forward	GCTTGAGGTGTAGATGTGTCC
	Reverse	CCCACGGGGCAAGATTTGAA
*IL-6*	Forward	GAACTCCTTCTCCACAAGCG
	Reverse	GCCTCTTTGCTGCTTTCACA
*IL-8*	Forward	TCCAAACCTTTCCACCCCAAA
	Reverse	TTTCTGTGTTGGCGCAGTGT
*TNF-α*	Forward	GGTCCTCTTCAAGGGCCAAG
	Reverse	TCACAGGGCAATGATCCCAA
*α-SMA*	Forward	GGCATTCACGAGACCACCTAC
	Reverse	CGACATGACGTTGTTGGCATAC
*Collagen Ⅰ*	Forward	GAGGGCCAAGACGAAGACATC
	Reverse	CAGATCACGTCATCGCACAAC
*GAPDH*	Forward	ATCCCATCACCATCTTCCAGGAG
	Reverse	CCTGCTTCACCACCTTCTTGATG

**Table 5:** List of primary antibodies (All antibodies we used are rabbit antibodies).

Antigens	Species	Dilution (IF)	Dilution (IHC)	Dilution (WB)	Supplier
CDK2	monoclonal			1:1000	CST, USA, Cat. 2546
CDK4	monoclonal			1:1000	CST, USA, Cat. 12790
Cyclin D1	monoclonal			1:1000	CST, USA, Cat. 2978
BAX	monoclonal			1:1000	Abclonal, CHN, Cat. A19684
BCL-2	monoclonal			1:1000	Abclonal, CHN, Cat. A19693
E-cadherin	polyclonal			1:1000	Abclonal, CHN, Cat. A3044
N-cadherin	monoclonal			1:1000	Abclonal, CHN, Cat. A19083
α-SMA	monoclonal	1:100		1:1000	Abclonal, CHN, Cat. A17910
Collagen Ⅰ	polyclonal	1:100		1:1000	Abclonal, CHN, Cat. A1352
TNF-α	polyclonal			1:1000	Abclonal, CHN, Cat. A0277
IL-6	polyclonal			1:1000	Abclonal, CHN, Cat. A0286
IL-8	polyclonal			1:1000	Abclonal, CHN, Cat. A2541
CXCL13	monoclonal	1:500	1:500	1:1000	Abcam, UK, Cat.ab112521
pAKT	monoclonal			1:1000	Abclonal, CHN, Cat. AP0637
AKT	monoclonal			1:1000	Abclonal, CHN, Cat. A17909
pERK1/2	monoclonal			1:1000	Abclonal, CHN, Cat. AP0472
ERK1/2	monoclonal			1:1000	Abclonal, CHN, Cat. A4782
pSTAT3	monoclonal			1:1000	Abclonal, CHN, Cat. AP0715
STAT3	polyclonal			1:1000	Abclonal, CHN, Cat. A1192
GAPDH	monoclonal			1:1000	Abclonal, CHN, Cat. AC001

**Table 6:** List of secondary antibodies used for Western blot.

Secondary Detection System Used	Host	Dilution Used	Supplier
Anti-Rabbit-IgG (H + L)-HRP	Goat	1:10000 (WB)	Sungene Biotech, Tianjin, China, Cat. #LK2001
		1:1000 (IHC)	
Anti-Rabbit IgG (H + L), F(ab’)2 fragment (Alexa Fluor^®^ (Irving, USA) 488 Conjugate)	Goat	1:50 (IF)	Cell Signaling Technology, Boston, USA, Cat. 4412
Hoechst 33342 (1 mg/mL) nucleic acid staining (DAPI)	-	1:750 (IF)	Molecular Probes/Invitrogen, Carlsbad, USA, Cat. A11007

## Data Availability

The data used to support the findings of this study are available from the corresponding author upon reasonable request.

## Supplementary Materials

The following supporting information can be downloaded at: https://www.mdpi.com/article/10.3390/ijms24010056/s1, Figure S1: rHuCXCL13 treatment had no effect on proliferation of WPMY-1 cells; Figure S2: Immunofluorescence staining of CXCL13 in BPH-1 and WPMY-1 cells; Figure S3: Immunofluorescence staining of α-SMA, Collagen I in WPMY-1 cells; Figure S4: The expression of CXCR5 in BPH-1 and WPMY-1 cells; Table S1: Sense sequences of siRNA.

## References

[1] Roehrborn C., Partin A.W., Dmochowski R.R., Kavoussi L.R., Partin A.W., Peters C.A. (2021). Benign prostatic hyperplasia: Etiology, pathophysiology, epidemiology and natural history. Campbell-Walsh Urology.

[2] Luo J., Dunn T., Ewing C., Sauvageot J., Chen Y., Trent J., Isaacs W. (2002). Gene expression signature of benign prostatic hyperplasia revealed by cDNA microarray analysis. Prostate.

[3] Middleton L.W., Shen Z., Varma S., Pollack A.S., Gong X., Zhu S., Zhu C., Foley J.W., Vennam S., Sweeney R.T. (2019). Genomic analysis of benign prostatic hyperplasia implicates cellular re-landscaping in disease pathogenesis. JCI Insight.

[4] Xiao H., Jiang Y., He W., Xu D., Chen P., Liu D., Liu J., Wang X., DiSanto M.E., Zhang X. (2020). Identification and functional activity of matrix-remodeling associated 5 (MXRA5) in benign hyperplastic prostate. Aging.

[5] Alturaiki W. (2022). Elevated Plasma Levels of CXCL13 Chemokine in Saudi Patients With Asthma Exacerbation. Cureus.

[6] Heinrichs M., Ashour D., Siegel J., Büchner L., Wedekind G., Heinze K.G., Arampatzi P., Saliba A.E., Cochain C., Hofmann U. (2021). The healing myocardium mobilizes a distinct B-cell subset through a CXCL13-CXCR5-dependent mechanism. Cardiovasc. Res..

[7] Zanetti C., Kumar R., Ender J., Godavarthy P.S., Hartmann M., Hey J., Breuer K., Weissenberger E.S., Minciacchi V.R., Karantanou C. (2021). The age of the bone marrow microenvironment influences B-cell acute lymphoblastic leukemia progression via CXCR5-CXCL13. Blood.

[8] Taniguchi T., Miyagawa T., Toyama S., Yamashita T., Nakamura K., Saigusa R., Ichimura Y., Takahashi T., Toyama T., Yoshizaki A. (2018). CXCL13 produced by macrophages due to Fli1 deficiency may contribute to the development of tissue fibrosis, vasculopathy and immune activation in systemic sclerosis. Exp. Dermatol..

[9] Perreau M., Suffiotti M., Marques-Vidal P., Wiedemann A., Levy Y., Laouénan C., Ghosn J., Fenwick C., Comte D., Roger T. (2021). The cytokines HGF and CXCL13 predict the severity and the mortality in COVID-19 patients. Nat. Commun..

[10] Kramer G., Mitteregger D., Marberger M. (2007). Is benign prostatic hyperplasia (BPH) an immune inflammatory disease?. Eur. Urol..

[11] Wang L., Yang J.R., Yang L.Y., Liu Z.T. (2008). Chronic inflammation in benign prostatic hyperplasia: Implications for therapy. Med. Hypotheses.

[12] Gandaglia G., Briganti A., Gontero P., Mondaini N., Novara G., Salonia A., Sciarra A., Montorsi F. (2013). The role of chronic prostatic inflammation in the pathogenesis and progression of benign prostatic hyperplasia (BPH). BJU Int..

[13] Penna G., Fibbi B., Amuchastegui S., Cossetti C., Aquilano F., Laverny G., Gacci M., Crescioli C., Maggi M., Adorini L. (2009). Human benign prostatic hyperplasia stromal cells as inducers and targets of chronic immuno-mediated inflammation. J. Immunol..

[14] Fibbi B., Penna G., Morelli A., Adorini L., Maggi M. (2010). Chronic inflammation in the pathogenesis of benign prostatic hyperplasia. Int. J. Androl..

[15] Isaacs J.T., Coffey D.S. (1989). Etiology and disease process of benign prostatic hyperplasia. Prostate Suppl..

[16] Li Y., Wang L., Yao L., Sun S., Shen L. (2019). CXCL13 promotes proliferation and autophagy of human mesenchymal stem cells through MAPK signaling pathway under hypoxic condition. Chin. J. Cell. Mol. Immunol..

[17] Da Z., Li L., Zhu J., Gu Z., You B., Shan Y., Shi S. (2016). CXCL13 Promotes Proliferation of Mesangial Cells by Combination with CXCR5 in SLE. J. Immunol. Res..

[18] Lisignoli G., Toneguzzi S., Piacentini A., Cristino S., Grassi F., Cavallo C., Facchini A. (2006). CXCL12 (SDF-1) and CXCL13 (BCA-1) chemokines significantly induce proliferation and collagen type I expression in osteoblasts from osteoarthritis patients. J. Cell. Physiol..

[19] Liu G., Wang Y., Jiang S., Sui M., Wang C., Kang L., Sun Y., Jiang Y. (2019). Suppression of lymphocyte apoptosis in spleen by CXCL13 after porcine circovirus type 2 infection and regulatory mechanism of CXCL13 expression in pigs. Vet. Res..

[20] Ma J.J., Jiang L., Tong D.Y., Ren Y.N., Sheng M.F., Liu H.C. (2018). CXCL13 inhibition induce the apoptosis of MDA-MB-231 breast cancer cells through blocking CXCR5/ERK signaling pathway. Eur. Rev. Med. Pharmacol. Sci..

[21] De Nunzio C., Kramer G., Marberger M., Montironi R., Nelson W., Schröder F., Sciarra A., Tubaro A. (2011). The controversial relationship between benign prostatic hyperplasia and prostate cancer: The role of inflammation. Eur. Urol..

[22] Wynn T.A. (2008). Cellular and molecular mechanisms of fibrosis. J. Pathol..

[23] Ma J., Gharaee-Kermani M., Kunju L., Hollingsworth J.M., Adler J., Arruda E.M., Macoska J.A. (2012). Prostatic fibrosis is associated with lower urinary tract symptoms. J. Urol..

[24] Rodriguez-Nieves J.A., Macoska J.A. (2013). Prostatic fibrosis, lower urinary tract symptoms, and BPH. Nat. Rev. Urol..

[25] Bushman W.A., Jerde T.J. (2016). The role of prostate inflammation and fibrosis in lower urinary tract symptoms. Am. J. Physiol. Ren. Physiol..

[26] Biswas S., Sengupta S., Roy Chowdhury S., Jana S., Mandal G., Mandal P.K., Saha N., Malhotra V., Gupta A., Kuprash D.V. (2014). CXCL13-CXCR5 co-expression regulates epithelial to mesenchymal transition of breast cancer cells during lymph node metastasis. Breast Cancer Res. Treat..

[27] Xie Y., Chen Z., Zhong Q., Zheng Z., Chen Y., Shangguan W., Zhang Y., Yang J., Zhu D., Xie W. (2021). M2 macrophages secrete CXCL13 to promote renal cell carcinoma migration, invasion, and EMT. Cancer Cell Int..

[28] Ammirante M., Shalapour S., Kang Y., Jamieson C.A., Karin M. (2014). Tissue injury and hypoxia promote malignant progression of prostate cancer by inducing CXCL13 expression in tumor myofibroblasts. Proc. Natl. Acad. Sci. USA.

[29] Singh S., Singh R., Sharma P.K., Singh U.P., Rai S.N., Chung L.W., Cooper C.R., Novakovic K.R., Grizzle W.E., Lillard J.W. (2009). Serum CXCL13 positively correlates with prostatic disease, prostate-specific antigen and mediates prostate cancer cell invasion, integrin clustering and cell adhesion. Cancer Lett..

[30] El-Haibi C.P., Singh R., Gupta P., Sharma P.K., Greenleaf K.N., Singh S., Lillard J.W. (2012). Antibody Microarray Analysis of Signaling Networks Regulated by Cxcl13 and Cxcr5 in Prostate Cancer. J. Proteom. Bioinform..

[31] Li F., Pascal L.E., Stolz D.B., Wang K., Zhou Y., Chen W., Xu Y., Chen Y., Dhir R., Parwani A.V. (2019). E-cadherin is downregulated in benign prostatic hyperplasia and required for tight junction formation and permeability barrier in the prostatic epithelial cell monolayer. Prostate.

[32] Chen W., Pascal L.E., Wang K., Dhir R., Sims A.M., Campbell R., Gasper G., DeFranco D.B., Yoshimura N., Wang Z. (2020). Differential impact of paired patient-derived BPH and normal adjacent stromal cells on benign prostatic epithelial cell growth in 3D culture. Prostate.

[33] Fraga C.H., True L.D., Kirk D. (1998). Enhanced expression of the mesenchymal marker, vimentin, in hyperplastic versus normal human prostatic epithelium. J. Urol..

[34] Alonso-Magdalena P., Brössner C., Reiner A., Cheng G., Sugiyama N., Warner M., Gustafsson J.A. (2009). A role for epithelial-mesenchymal transition in the etiology of benign prostatic hyperplasia. Proc. Natl. Acad. Sci. USA.

[35] Slabáková E., Pernicová Z., Slavíčková E., Staršíchová A., Kozubík A., Souček K. (2011). TGF-β1-induced EMT of non-transformed prostate hyperplasia cells is characterized by early induction of SNAI2/Slug. Prostate.

[36] Hu S., Yu W., Lv T.J., Chang C.S., Li X., Jin J. (2014). Evidence of TGF-β1 mediated epithelial-mesenchymal transition in immortalized benign prostatic hyperplasia cells. Mol. Membr. Biol..

[37] Broster S.A., Kyprianou N. (2015). Epithelial-mesenchymal transition in prostatic disease. Future Oncol..

[38] Zhao Q., Guo J., Wang G., Bi Y., Cheng X., Liao Y., Jin S., Li L., Guo Y., Pan L. (2021). CXCL13 promotes intestinal tumorigenesis through the activation of epithelial AKT signaling. Cancer Lett..

[39] Zheng Z., Cai Y., Chen H., Chen Z., Zhu D., Zhong Q., Xie W. (2018). CXCL13/CXCR5 Axis Predicts Poor Prognosis and Promotes Progression Through PI3K/AKT/mTOR Pathway in Clear Cell Renal Cell Carcinoma. Front. Oncol..

[40] Zhao J., Chen S., Yang C., Zhou M., Yang T., Sun B., Zhu J., Zhang H., Lu Q., Li L. (2022). Activation of CXCL13/CXCR5 axis aggravates experimental autoimmune cystitis and interstitial cystitis/bladder pain syndrome. Biochem. Pharmacol..

[41] Alvarez E., Piccio L., Mikesell R.J., Klawiter E.C., Parks B.J., Naismith R.T., Cross A.H. (2013). CXCL13 is a biomarker of inflammation in multiple sclerosis, neuromyelitis optica, and other neurological conditions. Mult. Scler..

[42] Bugatti S., Manzo A., Vitolo B., Benaglio F., Binda E., Scarabelli M., Humby F., Caporali R., Pitzalis C., Montecucco C. (2014). High expression levels of the B cell chemoattractant CXCL13 in rheumatoid synovium are a marker of severe disease. Rheumatology.

[43] Mack M. (2018). Inflammation and fibrosis. Matrix Biol. J. Int. Soc. Matrix Biol..

[44] Vuga L.J., Tedrow J.R., Pandit K.V., Tan J., Kass D.J., Xue J., Chandra D., Leader J.K., Gibson K.F., Kaminski N. (2014). C-X-C motif chemokine 13 (CXCL13) is a prognostic biomarker of idiopathic pulmonary fibrosis. Am. J. Respir. Crit. Care Med..

[45] Djavan B., Lin V., Seitz C., Kramer G., Kaplan P., Richier J., Marberger M., McConnell J.D. (1999). Elastin gene expression in benign prostatic hyperplasia. Prostate.

[46] Cantiello F., Cicione A., Salonia A., Autorino R., Tucci L., Madeo I., Damiano R. (2013). Periurethral fibrosis secondary to prostatic inflammation causing lower urinary tract symptoms: A prospective cohort study. Urology.

[47] Bauman T.M., Nicholson T.M., Abler L.L., Eliceiri K.W., Huang W., Vezina C.M., Ricke W.A. (2014). Characterization of fibrillar collagens and extracellular matrix of glandular benign prostatic hyperplasia nodules. PLoS ONE.

[48] Pinsky P.F., Kramer B.S., Crawford E.D., Grubb R.L., Urban D.A., Andriole G.L., Chia D., Levin D.L., Gohagan J.K. (2006). Prostate volume and prostate-specific antigen levels in men enrolled in a large screening trial. Urology.

[49] Mochtar C.A., Kiemeney L.A., van Riemsdijk M.M., Barnett G.S., Laguna M.P., Debruyne F.M., de la Rosette J.J. (2003). Prostate-specific antigen as an estimator of prostate volume in the management of patients with symptomatic benign prostatic hyperplasia. Eur. Urol..

[50] Bosch J.L., Bohnen A.M., Groeneveld F.P. (2004). Validity of digital rectal examination and serum prostate specific antigen in the estimation of prostate volume in community-based men aged 50 to 78 years: The Krimpen Study. Eur. Urol..

[51] Putra I.B., Hamid A.R., Mochtar C.A., Umbas R. (2016). Relationship of age, prostate-specific antigen, and prostate volume in Indonesian men with benign prostatic hyperplasia. Prostate Int..

[52] Jensen E.C. (2013). Quantitative analysis of histological staining and fluorescence using ImageJ. Anat. Rec..

