# Supplementary material for: Changes in the Expression and Functional Activities of C-X-C Motif Chemokine Ligand 13 (CXCL13) in Hyperplastic Prostate
Source: Int J Mol Sci. 2022 Dec 21;24(1):56. doi: 10.3390/ijms24010056 (PMC9820459; doi:10.3390/ijms24010056)
Supplement: Supplementary file 1 [file ijms-24-00056-s001.zip › ijms-2031768-supplementary.pdf]

Supplementary

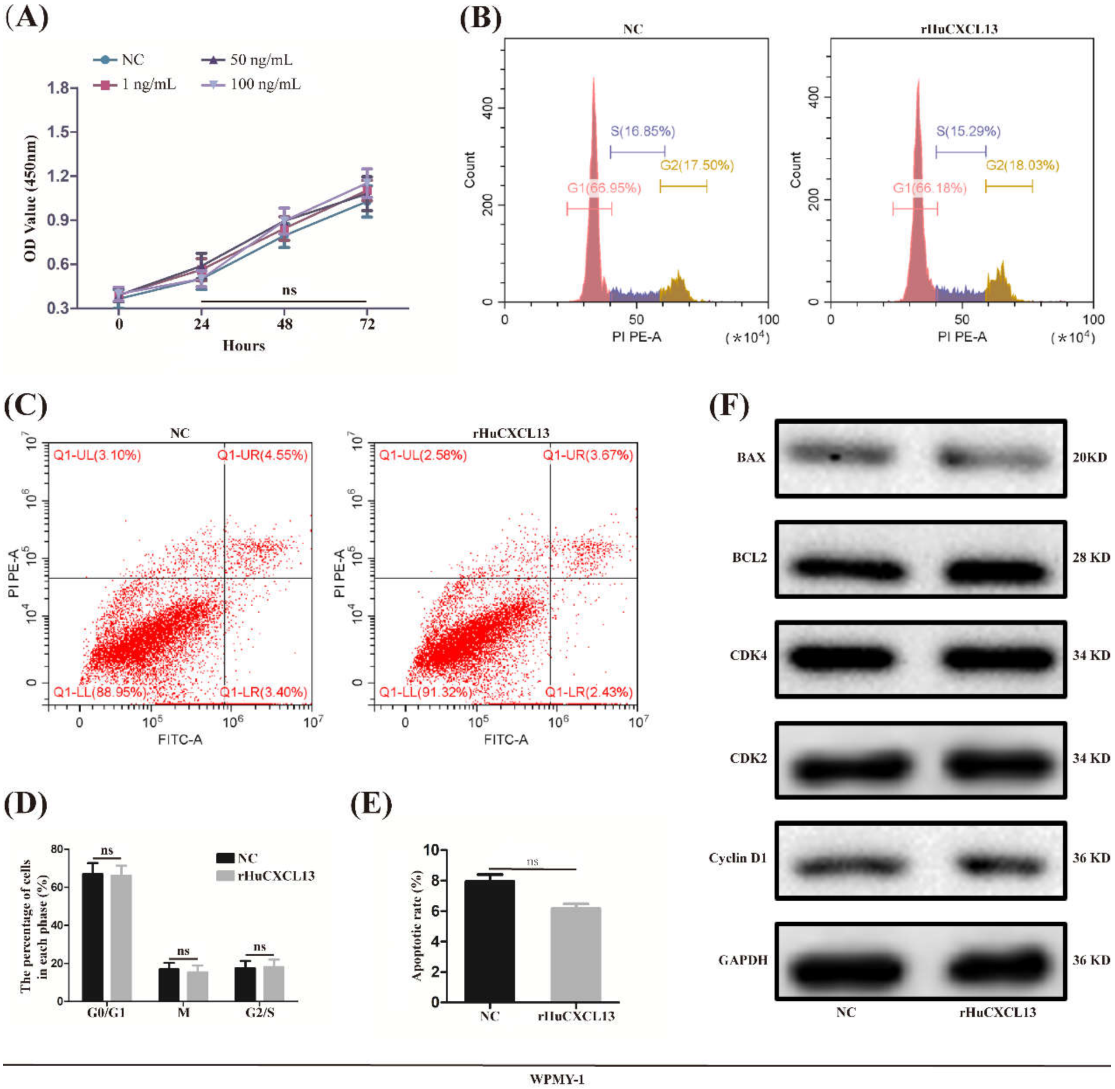

Figure S1. rHuCXCL13 treatment had no effect on proliferation of WPMY-1 cells. (**A**) CCK8 assay of WPMY-1 cells. (**B,C**) Flow cytometry analysis of cell cycle and cell apoptosis in WPMY-1 cells. (**D,E**) Statistical analysis of percentages (%) of cells at each stage and apoptotic rate (%) in WPMY-1 cells. (**F**) Western-blot assay of cell cycle- and apoptosis-related proteins in WPMY-1 cells. ns means no significant difference.

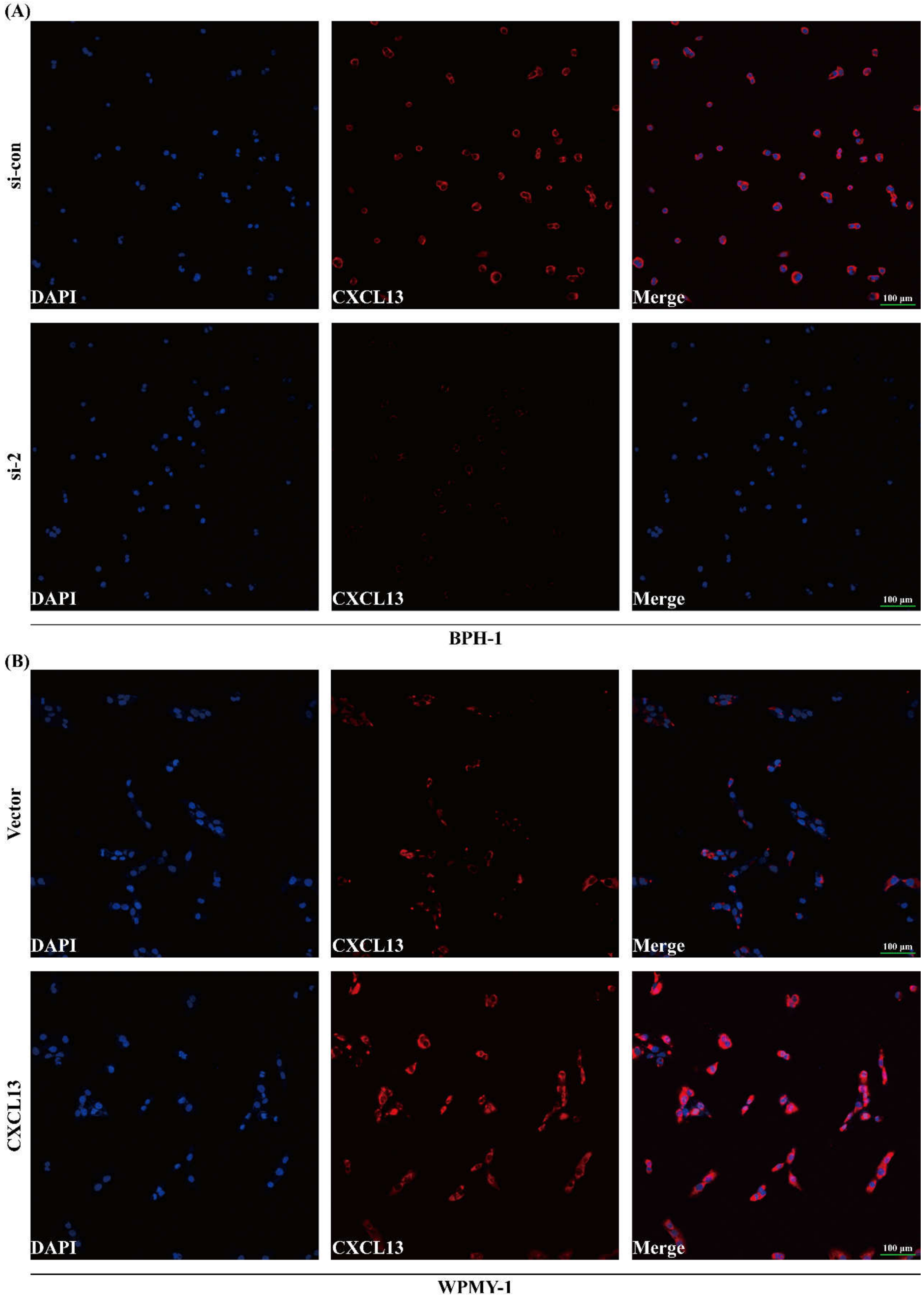

Figure S2. Immunofluorescence staining of CXCL13 in BPH-1 and WPMY-1 cells. (**A**) Immunofluorescence staining of CXCL13 in BPH-1 cells after CXCL13 knockdown. (**B**) Immunofluorescence staining of CXCL13 in WPMY-1 cells after CXCL13 overexpression. DAPI

(blue) indicates nuclear staining and Cy3-immunofluorescence (red) indicates CXCL13 protein staining. Representative graphs are shown. ALL scale bars are 100 μm.

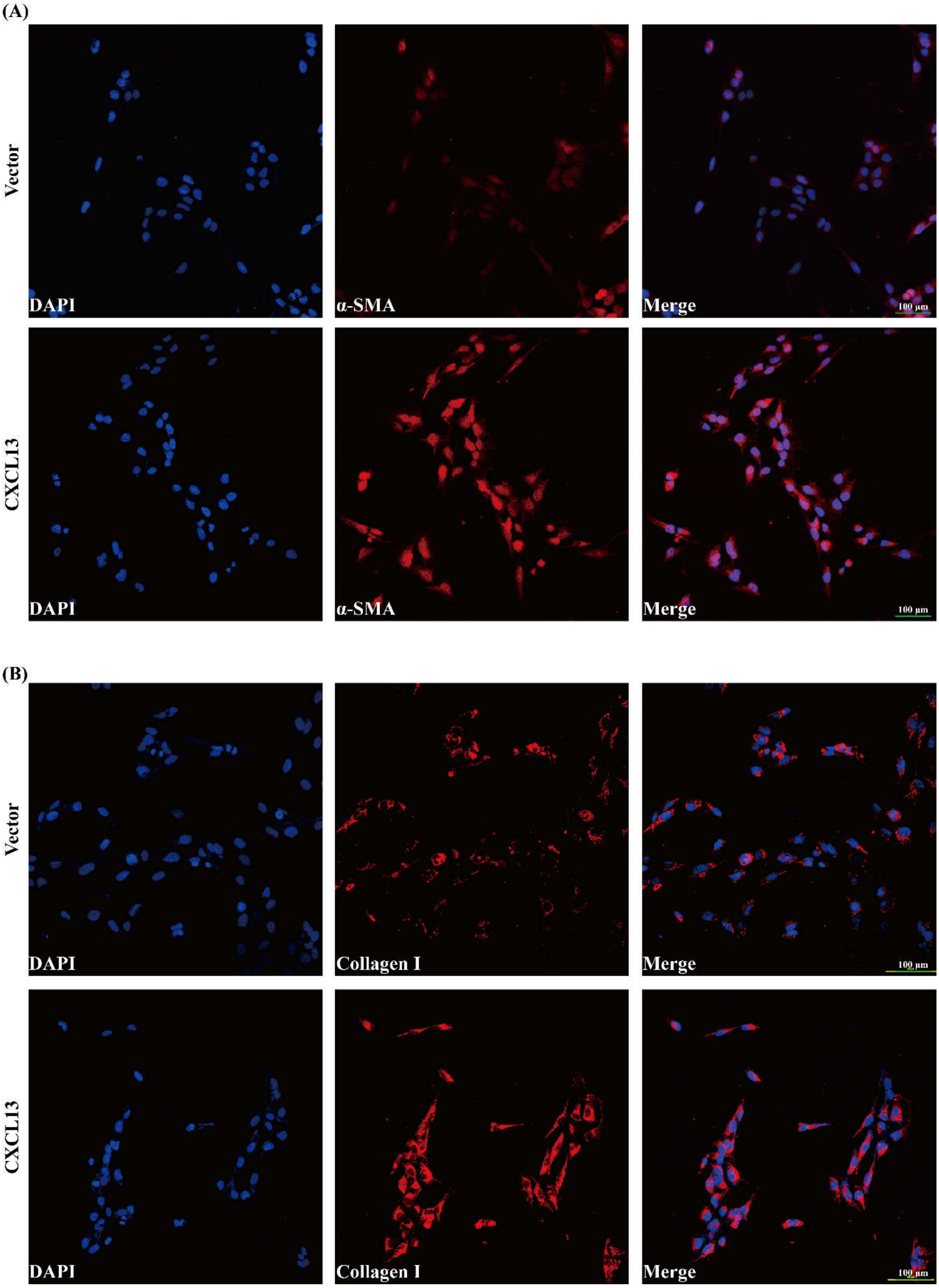

Figure S3. Immunofluorescence staining of α-SMA, Collagen I in WPMY-1 cells. (**A**,**B**) Immunofluorescence staining of α-SMA, Collagen I in WPMY-1 cells after CXCL13

overexpression. DAPI (blue) indicates nuclear staining and Cy3-immunofluorescence (red) indicates α-SMA, Collagen I protein staining. Representative graphs are shown. ALL scale bars are 100 μm.

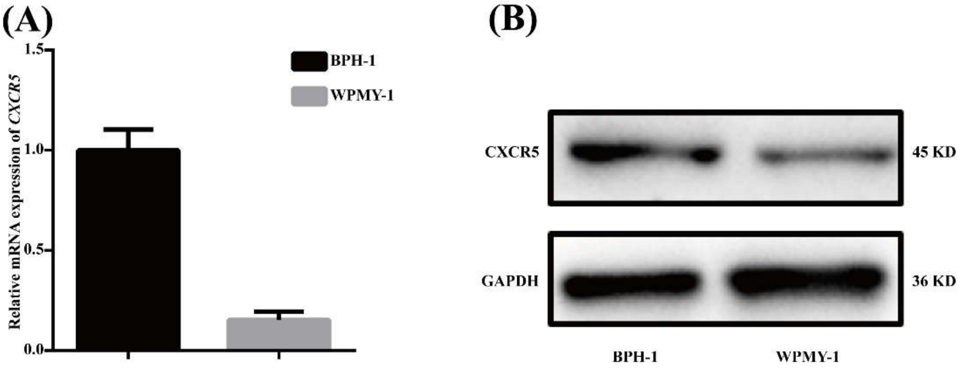

Figure S4. The expression of CXCR5 in BPH-1 and WPMY-1 cells. (**A**) The mRNA expression of CXCL13 in BPH-1 and WPMY-1 cells. (**B**) The protein expression of CXCL13 in BPH-1 and WPMY-1 cells.

Table S1. Sense sequences of siRNA.

		**Sense Sequences (5′ to 3′)**
si-con	Forward	UUCUCCGAACGUGUCACGUTT
	Reverse	ACGUGACACGUUCGGAGAATT
si-1	Forward	CCAAGAGAGCUCAGUCUUUTT
	Reverse	AAAGACUGAGCUCUCUUGGTT
si-2	Forward	CCCUCAAGCUGAAUGGAUATT
	Reverse	UAUCCAUUCAGCUUGAGGGTT
